# Maternal BMI and allergy in children until 3 years of age (JECS)

**DOI:** 10.1016/j.jacig.2022.02.003

**Published:** 2022-03-10

**Authors:** Daisuke Hayashi, Emiko Noguchi, Kazushi Maruo, Monami Hara, Shoji F. Nakayama, Hidetoshi Takada, the Japan Environment and Children’s Study Group, Michihiro Kamijima, Shin Yamazaki, Yukihiro Ohya, Reiko Kishi, Nobuo Yaegashi, Koichi Hashimoto, Chisato Mori, Shuichi Ito, Zentaro Yamagata, Hidekuni Inadera, Takeo Nakayama, Hiroyasu Iso, Masayuki Shima, Youichi Kurozawa, Narufumi Suganuma, Koichi Kusuhara, Takahiko Katoh

**Affiliations:** a Japan Environment and Children’s Study Program Office, National Institute for Environmental Studies, Tsukuba, Japan; b Department of Medical Genetics, Faculty of Medicine, University of Tsukuba, Tsukuba, Japan; d Department of Biostatistics, Faculty of Medicine, University of Tsukuba, Tsukuba, Japan; f Department of Child Health, Faculty of Medicine, University of Tsukuba, Tsukuba, Japan; c Department of Pediatrics, Tsukuba Medical Center Hospital, Tsukuba, Japan; e Department of Pediatrics, University of Tsukuba Hospital, Tsukuba, Japan

**Keywords:** Atopic dermatitis, asthma, cow’s milk allergy, egg allergy, food allergy, prepregnancy maternal body mass index

## Abstract

**Background:**

Maternal prepregnancy body mass index (BMI) may influence allergic diseases in the children who are the product of those pregnancies.

**Objective:**

The purpose of our study was to investigate the association between mothers' prepregnancy BMI and the risk of physician-diagnosed asthma, food allergy (FA), and atopic dermatitis (AD) in their children during the first 3 years of life.

**Methods:**

Data on mothers' prepregnancy BMI and physician-diagnosed asthma, FA, and AD in their children until the age of 3 years were obtained from the Japan Environment and Children’s Study, a nationwide birth cohort study that has recruited 103,099 pregnant women between 2011 and 2014. Logistic regression analysis was used to analyze the results.

**Results:**

We analyzed 67,204 mother-child pairs with available information on physician-diagnosed allergic diseases. The risk of asthma was significantly higher in children born to overweight mothers (adjusted OR [aOR] =1.17 [95% CI = 1.07-1.28]) and obese mothers (aOR = 1.28 [95% CI = 1.08-1.50]), whereas the risk of FA, cow’s milk allergy, and egg allergy decreased significantly in children born to overweight mothers (aOR = 0.84 [95% CI = 0.76-0.92]; aOR = 0.78 [95% CI = 0.64-0.93]; and aOR = 0.83 [95% CI = 0.74-0.94]) and obese mothers (aOR = 0.81 [95% CI = 0.67-0.97]; aOR = 0.58 [95% CI = 0.36-0.87]; and aOR = 0.73 [95% CI = 0.56-0.93]) compared with in children born to normal weight mothers, respectively. Associations between AD and maternal BMI were not detected.

**Conclusion:**

Our study showed that an increase in mothers' prepregnancy BMI was associated with an increase in asthma prevalence and a decrease in FA prevalence in their children. Further studies are needed to reveal the mechanisms associated with maternal BMI and pediatric allergic diseases.

Allergic diseases are common and complex conditions, and both genetic and environmental factors are involved in their development.^1^ Parental allergic diseases are strongly associated with the development of childhood allergies; for instance, maternal bronchial asthma is related to the development of childhood asthma,^2^ and a parental history of eczema increases the risk of atopic dermatitis (AD) during childhood.^3^ In addition to these individual predispositions, environmental factors during the fetal and postnatal periods, such as diet and other components of lifestyle, influence their development.^4^^,^^5^ Epigenetic modifications of genes through these environmental factors influence the development and persistence of allergic diseases.^6^

The parental environment is an important factor that affects the health of children after birth in various ways. Many studies have been conducted to elucidate the relationship between the fetal environment and childhood diseases based on the developmental origins of the concept of health and disease.^7^ In regard to allergic diseases, Zhong et al reported that maternal antibiotic administration during pregnancy increased the risk of childhood asthma and AD,^8^ and Neuman et al reported that maternal smoking during pregnancy increased the risk of asthma in children.^9^ The mother’s physique may also alter the fetal environment and affect the development of allergic diseases in children.

Increased body fat mass is thought to be related to chronic inflammation and has been shown to increase the production of proinflammatory cytokines, such as C-reactive protein, which is an indicator of inflammation.^10^, ^11^, ^12^, ^13^ Adipokines are regulatory peptides that are secreted from adipose tissues and affect many organs.^14^^,^^15^ Some adipokines have a proinflammatory effect, whereas other adipokines have an anti-inflammatory effect. Leptin, a notable adipokine secreted from adipose cells,^16^ is a proinflammatory cytokine associated with asthma.^17^, ^18^, ^19^, ^20^ Obesity increases leptin secretion, which induces airway inflammation and may lead to the development of asthma,^17^, ^18^, ^19^, ^20^ whereas the level of adiponectin, which has anti-inflammatory effects, has been reported to decrease in obese people.^21^ A previous study using a mouse model experiment with ovalbumin sensitization showed that serum adiponectin levels were decreased during pulmonary allergic reactions and treatment with adiponectin attenuated allergic airway inflammation.^22^ These findings suggest that maternal body fat mass can be an important factor in the development of allergic diseases in the children.

In Europe and the United States, it has been reported that the risk of childhood asthma is correlated with the mother’s prepregnancy body mass index (BMI).^23^^,^^24^ However, the relationship between the mother’s prepregnancy BMI and other allergic diseases, such as food allergy (FA) and AD, has not been studied extensively.^25^ Asians tend to be thinner than Westerners, and a recently conducted meta-analysis revealed that the proportion of mothers with a prepregnancy BMI less than 18.5 kg/m^2^ was higher among Asians than among Westerners and the proportion of those with a prepregnancy BMI of 28 to 30 kg/m^2^ was lower among Asians than among Westerners.^26^ These anthropometric differences are influenced by both genetic and environmental factors, and the effect of the mothers’ physique on the development of allergic diseases in children may vary among different races and populations.^27^ However, few large-scale studies of non-White races have been conducted to assess the effects of a mothers’ physique on the development of allergic diseases in their children.^28^ Investigating the relationship between Asian mothers' prepregnancy BMI and allergic diseases in their children may provide novel insights into the appropriate management of BMI during pregnancy.

The present study explored the relationship between mothers' prepregnancy BMI and allergic diseases, including asthma, FA, and AD, in the children resulting from those pregnancies during the first 3 years of the children's lives. The analysis used data from the Japan Environment and Children’s Study (JECS), which is an ongoing nationwide cohort study that has recruited more than 103,000 pregnant women and investigated allergic diseases in children.^29^

## Methods

### Participants

JECS is a nationwide birth cohort study that recruited pregnant women from January 2011 to March 2014.^30^^,^^31^ JECS is funded by the Ministry of the Environment of Japan and has recruited 103,099 pregnant women at 15 regional centers located in a wide geographic area in Japan. The eligibility criteria for JECS are as follows: (1) participants should reside in the study areas at the time of recruitment and are expected to reside continually in Japan for the foreseeable future; (2) their expected delivery date should be between August 1, 2011, and mid-2014; and (3) they should be capable of participating in the study without difficulty.^30^ JECS participants are considered to represent the Japanese general population because the characteristics of mothers and children are comparable to the data comprising the vital statistics of Japan.^31^ The JECS protocol was reviewed and approved by Institutional Review Board on Epidemiological Studies of the Ministry of the Environment of Japan and by the ethics committees of all participating institutions. Written informed consent was obtained from all mothers and fathers participating in JECS, and data were collected by using questionnaires answered by parents and by reviewing their medical records, documented by physicians, midwives and/or nurses, and/or research coordinators. We used the jecs-ta-20190930 data set, which was released in October 2019.

**Fig 1:**
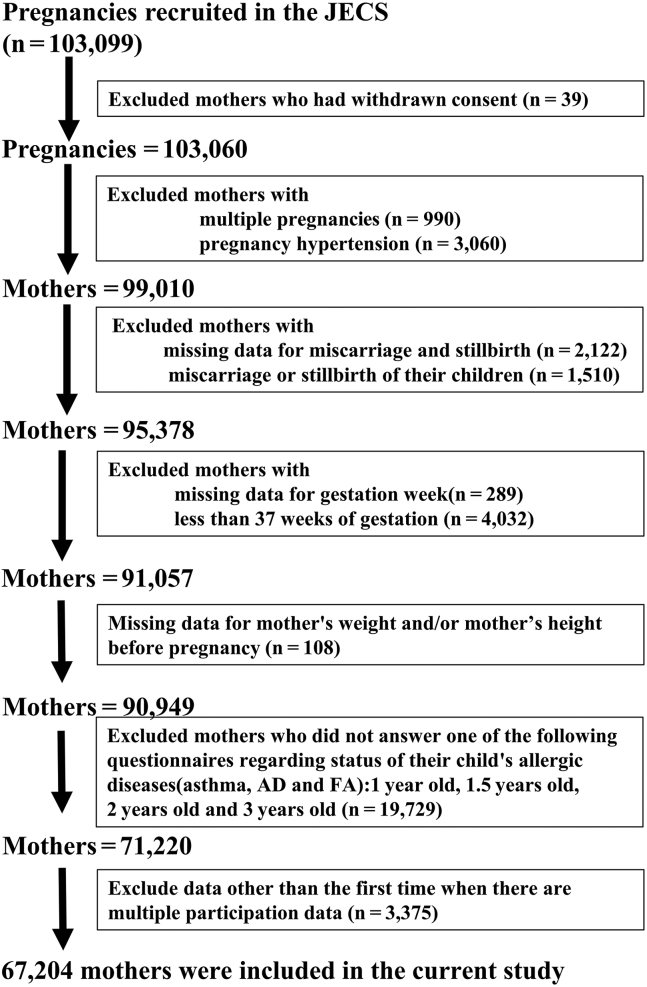
Flow diagram of the study.

Of the pregnant women whose 103,099 pregnancies were examined in JECS, 39 were excluded because they withdrew their consent and 990 and 3,060 were excluded owing to multiple pregnancies and gestational hypertension, respectively. Among the remaining 99,010 mothers, 2,122 and 1,510 were excluded owing to missing data on miscarriage and stillbirth, respectively. Among the 95,378 mothers without gestational hypertension, we excluded 289 and 4,032 mothers on account of missing data on gestation week or less than 37 weeks of gestation, respectively. Moreover, 108 mothers were excluded because of missing data on the mother's weight, height, or both before pregnancy. We then excluded data obtained from 19,729 mothers who did not answer 1 of the subsequent questionnaires regarding the status of their child’s allergic diseases (asthma, FA, and AD) at the ages of 1, 1.5, 2, and 3 years. Regarding multiple pieces of data on the same participant, 3,375 pieces were excluded (ie, all except for the first piece). Finally, 67,204 mothers were included in the analysis (Fig 1).

### Exposures

The prepregnancy height and weight of the mothers was obtained from their medical records transcripts or the questionnaire. Prepregnancy BMI (in kg/m^2^) was calculated and divided into 4 categories: (underweight, <18.5 kg/m^2^; normal weight, 18.5 to <25 kg/m^2^; overweight, 25 to <30 kg/m^2^; and obese, ≥30 kg/m^2^) according to the World Health Organization classification.^26^

### Outcomes

The status of childhood asthma, FA, cow’s milk allergy (CMA), egg allergy (EA), and AD were obtained from questionnaires answered by the mothers when their children were 1, 1.5, 2, and 3 years old. We defined physician-diagnosed asthma, FA, and AD as asthma, FA, and AD that were reported by the mothers to have been diagnosed by a physician at least once. FA was defined as including both CMA and EA. Children were considered to have CMA or EA when they satisfied both of the following criteria at least once: (1) met the criteria of physician-diagnosed FA as described earlier in this paragraph and (2) experienced an allergic reaction after ingesting milk or egg.

### Covariates

We tested 2 covariate models. In model 1, the following factors related to the prenatal environment were adjusted for analysis: maternal age, smoking during pregnancy, maternal allergic diseases (maternal asthma, maternal FA, and maternal AD), maternal education, and child’s sex based on previous studies.^23^^,^^25^^,^^32^, ^33^, ^34^ In model 2, factors related to the feeding of children after birth were added to the covariates used in model 1 and were adjusted for analysis; the covariates added to model 1 were formula feeding until 1 month after birth and the start of complementary feeding at 6 months after birth.

### Statistical analysis

To assess the difference between each BMI category, we compared the characteristics of the participating mothers and children in each prepregnancy BMI group by using ANOVA for continuous variables and the chi-squared test for categoric variables (Table I). For association analysis between mothers' prepregnancy BMI and the outcomes of physician-diagnosed asthma, FA, CMA, EA, and AD in their children, we used binominal logistic regression analysis to calculate odds ratios (ORs) and 95% CIs, with mothers having a BMI of 18.5 to less than 25 kg/m^2^ serving as a reference. All statistical analyses were conducted by using R version 3.6.3 (R Foundation for Statistical Computing, Vienna, Austria).

**Table I:** Characteristics of the participants

Characteristic	Prepregnancy maternal BMI, kg/m^2^				
	All (n = 67,204)	<18.5 (n = 10,918)	18.5 to <25 (n = 50,113)	25 to <30 (n = 4,907)	≥30 (n = 1,266)
Background of mothers					
Age at delivery (y), mean (SD)	31.48 (4.88)	30.67 (4.88)	31.58 (4.84)	32.08 (4.99)	32.07 (4.82)
Delivery by cesarean section, no. (%)					
No	55,627 (83.0)	9,452 (86.8)	41,668 (83.4)	3,655 (74.6)	852 (67.5)
Yes	11,407 (17.0)	1,438 (13.2)	8,313 (16.6)	1,245 (25.4)	411 (32.5)
Education (y), no. (%)					
<10	2,334 (3.5)	418 (3.9)	1,560 (3.1)	256 (5.3)	100 (8.0)
10-12	19,681 (29.5)	3,166 (29.2)	14,244 (28.7)	1,748 (35.9)	523 (42.0)
≥13	44,612 (67.0)	7,241 (66.9)	33,889 (68.2)	2,859 (58.8)	623 (50.0)
Income (million yen), no. (%)					
<4	24,006 (38.3)	4,056 (40.2)	17,373 (37.1)	2,001(43.8)	576 (49.3)
4-5	21,125 (33.7)	3,226 (32.0)	16,019 (34.2)	1,511 (33.1)	369 (31.6)
6-7	10,432 (16.6)	1,595 (15.8)	8,029 (17.1)	662 (14.5)	146 (12.5)
≥8	7,114 (11.4)	1,203 (11.9)	5,442 (11.6)	391 (8.6)	78 (6.7)
Smoking in pregnancy, no. (%)					
No	64,725 (96.9)	10,461 (96.5)	48,415 (97.2)	4,664 (95.6)	1,185 (94.5)
Yes	2,061 (3.1)	383 (3.5)	1,394 (2.8)	215 (4.4)	69 (5.5)
Asthma, no. (%)					
No	59,885 (89.5)	9,820 (90.4)	44,792 (89.8)	4,233 (86.6)	1,040 (82.8)
Yes	7,009 (10.5)	1,039 (9.6)	5,099 (10.2)	655 (13.4)	216 (17.2)
FA, no. (%)					
No	63,712 (95.2)	10,335 (95.2)	47,554 (95.3)	4,637 (94.9)	1186 (94.4)
Yes	3,182 (4.8)	524 (4.8)	2,337 (4.7)	251 (5.1)	70 (5.6)
AD, no. (%)					
No	56,127 (83.9)	9,092 (83.7)	41,837 (83.9)	4,117 (84.2)	1,081 (86.1)
Yes	10,767 (16.1)	1,767 (16.3)	8,054 (16.1)	771 (15.8)	175 (13.9)
Background of children					
Child sex, no. (%)					
Male	34,248 (51.0)	5,584 (51.1)	25,436 (50.8)	2,569 (52.4)	659 (47.9)
Female	32,956 (49.0)	5,334 (48.9)	24,677 (49.2)	2,338 (47.6)	607 (47.9)
Gestational age (wk), mean (SD)	39.07 (1.14)	38.99 (1.11)	39.08 (1.14)	39.07 (1.21)	39.09 (1.25)
Physician diagnosed asthma, no. (%)					
No	59,615 (88.7)	9,729 (89.1)	44,536 (88.9)	42,69 (87.0)	1,081 (85.4)
Yes	7,589 (11.3)	1,189 (10.9)	5,577 (11.1)	638 (13.0)	185 (14.6)
Physician diagnosed FA, no. (%)					
No	58,472 (87.0)	9,509 (87.1)	43,442 (86.7)	4,380 (89.3)	1,141 (90.1)
Yes	8,732 (13.0)	1,409 (12.9)	6,671 (13.3)	527 (10.7)	125 (9.9)
CMA, no. (%)					
No	64,938 (96.6)	10,578 (96.9)	48,327 (96.4)	4,788 (97.6)	1,245 (98.3)
Yes	2,266 (3.4)	340 (3.1)	1,786 (3.6)	119 (2.4)	21 (1.7)
EA, no. (%)					
No	61,867 (92.1)	10,069 (92.2)	46,006 (91.8)	4,593 (93.6)	1,199 (94.7)
Yes	5,337 (7.9)	849 (7.8)	4,107 (8.2)	314 (6.4)	67 (5.3)
Physician-diagnosed AD, no. (%)					
No	59,484 (88.5)	9,645 (88.3)	44,346 (88.5)	4,371 (89.1)	1,122 (88.6)
Yes	7,720 (11.5)	1,273 (11.7)	5,767 (11.5)	536 (10.9)	144 (11.4)

## Results

### Participant characteristics

The baseline characteristics of the mothers and children who participated in our study are shown in Table I. In regard to prepregnancy maternal BMI, 10,918 participants (16.2%) were underweight (<18.5 kg/m^2^), 50,113 (74.6%) were of normal weight (18.5 to <25 kg/m^2^), 4,907 (7.3%) were overweight (25 to <30 kg/m^2^), and 1,266 (1.9%) were obese (≥30 kg/m^2^). There were 7,589 children (11.3%) with physician-diagnosed asthma before 3 years of age, 8,732 children (13.0%) with FA, 2,266 (3.4%) with CMA, 5,337 (7.9%) with EA, and 7,720 (11.5%) with AD. The numbers of children who were analyzed in each covariate model are presented in Table E1 (available in this article’s Online Repository at www.jaci-global.org).

### Asthma

**Fig 2:**
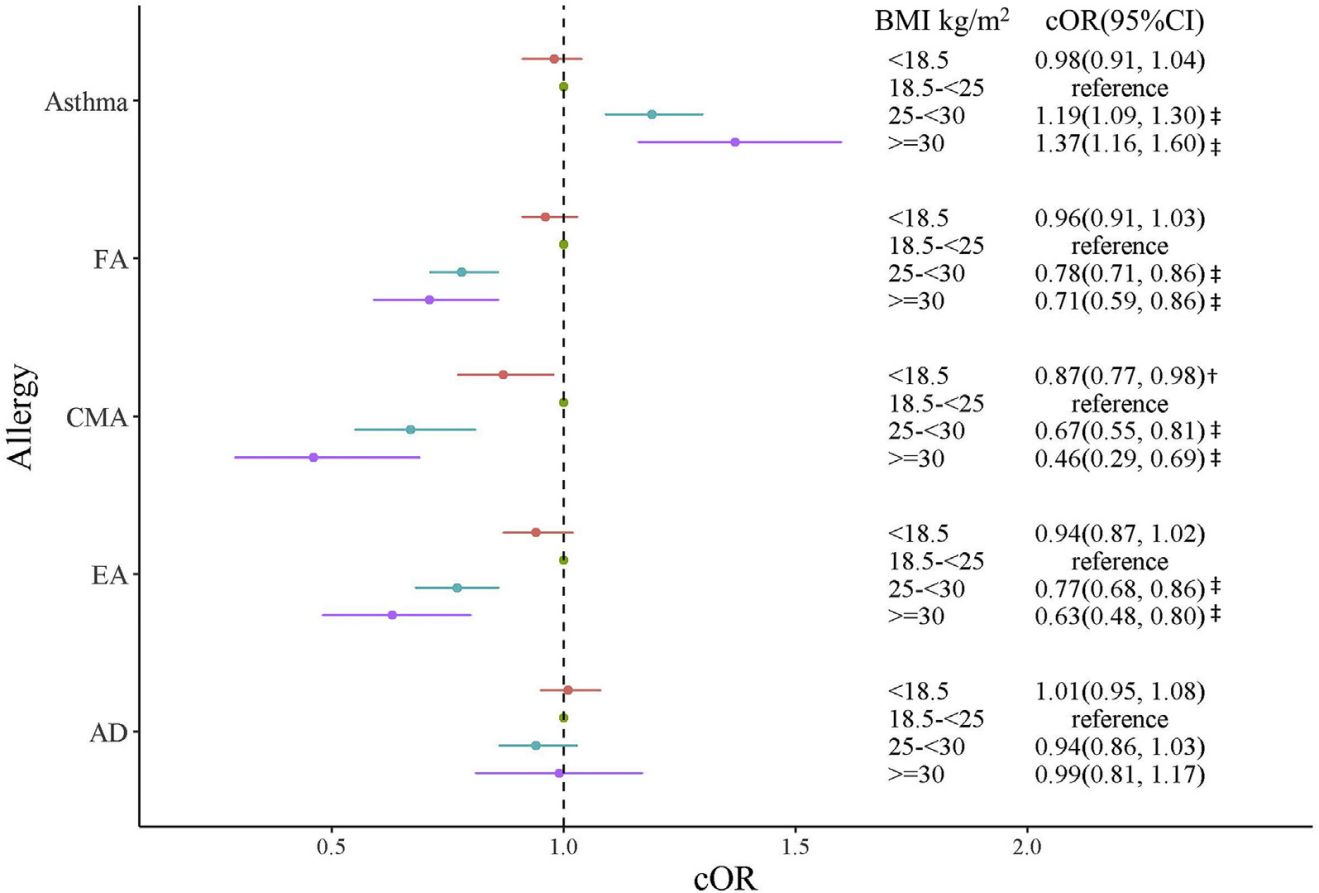
Relationship between mothers' prepregnancy BMI and allergic disease in their children without adjustment. ^†^*P* < .05; ^‡^*P* < .001.

**Fig 3:**
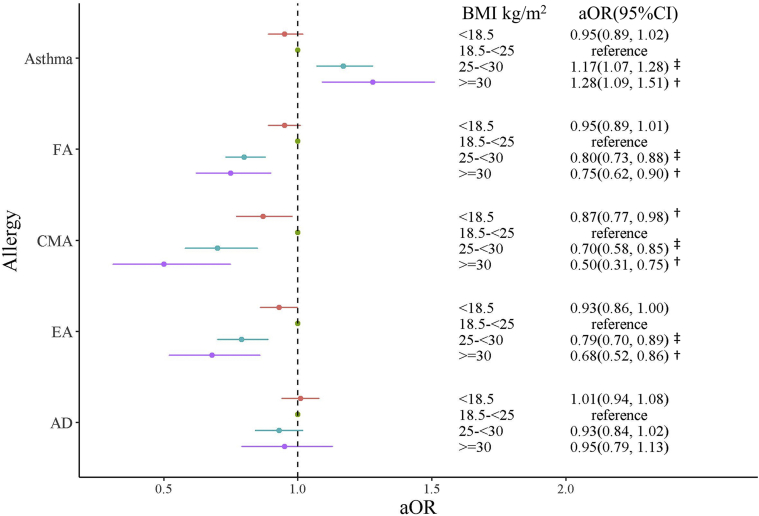
Relationship between maternal BMI and FA in the children, adjusted with cofounding factors (model 1). Adjusted for maternal age, smoking in pregnancy, maternal allergic diseases, child’s sex, and maternal education. ^†^*P* < .05; ^‡^*P* < .001.

**Fig 4:**
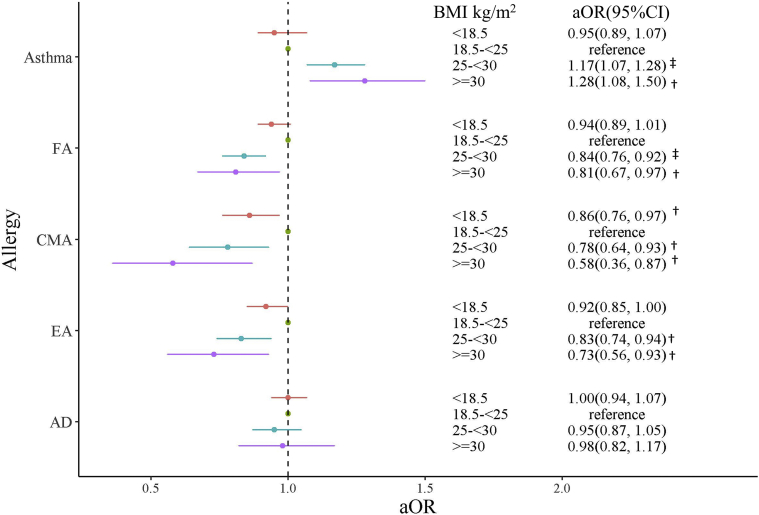
Relationship of maternal BMI and FA in the children to child feeding pattern (model 2) Adjusted for maternal age, smoking in pregnancy, maternal allergic diseases, child’s sex, maternal education, formula feeding at 1 month after birth, and feeding of complementary food at 6 months after birth. ^†^*P* < .05; ^‡^*P* < .001.

When the nonadjusted analysis and adjusted models 1 and 2 were used, the odds of physician-diagnosed asthma in children aged 3 years or younger were significantly increased in children of overweight mothers (crude OR [cOR] = 1.19 [95% CI = 1.09-1.30]; *P < .*001 [Fig 2]; adjusted OR [aOR] = 1.17 [95% CI = 1.07-1.28]; *P < .*001 [Fig 3]; and aOR = 1.17 [95% CI = 1.07-1.28]; *P < .*001 [Fig 4]) and children of obese mothers (cOR = 1.37 [95% CI = 1.16-1.60]; *P < .*001 [Fig 2]; aOR = 1.28 [95% CI = 1.09-1.51]; *P = .*003 [Fig 3]; and aOR = 1.28 [95% CI = 1.08-1.50]; *P = .*003 [Fig 4]).

### FA

When nonadjusted analysis and adjusted models 1 and 2 were used, the odds of physician-diagnosed FA were significantly decreased in children of overweight mothers (cOR = 0.78 [95% CI = 0.71-0.86]; *P < .*001 [Fig 2]; aOR = 0.80 [95% CI = 0.73-0.88]; *P < .*001 [Fig 3]; and aOR = 0.84 [95% CI = 0.76-0.92]; *P < .*001 [Fig 4]) and children of obese mothers (cOR = 0.71 [95% CI = 0.59-0.86]; *P < .*001 [Fig 2]; aOR = 0.75 [95% CI = 0.62-0.90]; *P = .*003 [Fig 3]; and aOR = 0.81 [95% CI = 0.67-0.97]; *P = .*028 [Fig 4]). When nonadjusted analysis and adjusted models 1 and 2 were used, the risk of CMA was significantly decreased in children of overweight mothers (cOR = 0.67 [95% CI = 0.55-0.81]; *P < .*001 [Fig 2]; aOR = 0.70 [95% CI = 0.58-0.85]; *P < .*001 [Fig 3]; and aOR = 0.78 [95% CI = 0.64-0.93]; *P = .*009 [Fig 4]) and children of obese mothers (cOR = 0.46 [95% CI = 0.29-0.69]; *P < .*001 [Fig 2]; aOR = 0.50 [95% CI = 0.31-0.75]; *P = .*02 [Fig 3]; and aOR = 0.58 [95% CI = 0.36-0.87]; *P = .*015 [Fig 4]). The risk of CMA was also decreased in children of underweight mothers (cOR = 0.87 [95% CI = 0.77-0.98]; *P = .*02 [Fig 2]; aOR = 0.87 [95% CI = 0.77-0.98]; *P* = .02 [Fig 3]; and aOR = 0.86 [95% CI = 0.76-0.97]; *P* = .013 [Fig 4]). With use of the nonadjusted analysis and adjusted models 1 and 2, the risk of EA was significantly decreased in children of mothers with a high prepregnancy BMI compared with that in children of mothers with a normal prepregnancy BMI (for overweight mothers, cOR = 0.77 [95% CI = 0.68-0.86]; *P < .*001 [Fig 2]; aOR = 0.79 [95% CI = 0.70-0.89]; *P < .*001 [Fig 3]; and aOR = 0.83 [95% CI = 0.74-0.94]; *P = .*003 [Fig 4], whereas for obese mothers, cOR = 0.63 [95% CI = 0.48-0.80]; *P < .*001 [Fig 2]; aOR = 0.68 [95% CI = 0.52-0.86]; *P = .*002 [Fig 3]; and aOR = 0.73 [95% CI = 0.56-0.93]; *P = .*013 [Fig 4]).

### AD

Associations between AD and prepregnancy maternal BMI with no adjustment (Fig 2) and with adjustment (Figs 3 and 4) were not detected.

## Discussion

In the present study, which was a large, nationwide birth cohort study, we found a relationship between maternal prepregnancy BMI and allergic diseases in the children resulting from those pregnancies. An increase in mothers' prepregnancy BMI was associated with an increased risk of childhood asthma and a decreased risk of FA in their children.

Previous studies have reported that increased mothers' prepregnancy BMI is associated with an increased risk of asthma and wheezing in their children.^23^^,^^25^^,^^35^, ^36^, ^37^, ^38^ Most previous studies were conducted in Europe and the United States, and the study populations included White, Hispanic, and Black ethnicities. Our results confirmed that obesity during pregnancy is associated with an increased risk of asthma development in children, suggesting that mothers' prepregnancy obesity and the risk of asthma in their children may be a universal phenomenon, independent of race. Elevated levels of inflammation-related factors such as IL-6, C-reactive protein, and TNF-α are found in obese pregnant women,^39^ which may contribute to the development of wheezing and asthma in children.

In the present study, the risk of FA in children decreased with higher prepregnancy BMI of their mothers. Kuma et al reported that there was no association between prepregnancy BMI of the mother and FA in the child.^25^ Their results may have differed from ours because of environmental and genetic differences, such as mixed races (59.1% African American and 21% Latino) and social factors. Chen et al showed that there is no relationship between maternal prepregnancy BMI and FA in Asian children^28^; however, their study included children between the ages of 3 and 14, with different criteria for BMI than in our study.

There are at least 2 possible reasons for the lower frequency of FA in children born to mothers with a higher prepregnancy BMI. The first possibility is early childhood exposure to formula and early initiation of complementary foods. Continuous use of formula from early infancy has been reported to reduce the frequency of milk allergies,^40^, ^41^, ^42^ and obese mothers have been reported to use formula more frequently.^43^, ^44^, ^45^ It has also been reported that obesity in pregnant mothers increases the risk of cesarean section because of complications associated with pregnancy and delivery, making breast-feeding more difficult.^46^ In addition, obese mothers tend to have larger breasts, which may interfere with the child's contact with the breast.^46^ These factors are thought to increase the frequency of formula use in obese mothers. In addition, the timing of initiation of complementary food influences the onset of food allergies, and mothers with a high BMI tend to start complementary food earlier than 6 months.^47^ Natsume et al reported that the introduction of egg to high-risk children at the age of 6 months reduced the frequency of EA,^48^ and Du Toit et al showed that the introduction of peanuts at the age of 4 to 11 months reduced the risk of peanut allergy.^49^ In the present study, the risk of FA in children was also decreased with higher prepregnancy BMI of the mother, even after adjustment for these factors in model 2; therefore, the association between the mother's BMI and the development of FA in children cannot be explained by the use of formula or the timing of complementary foods.

In our study, we found no association between prepregnancy BMI and AD in children. Harpsøe et al reported that there was no significant association between maternal prepregnancy BMI or weight gain during pregnancy and atopic eczema.^23^ Drucker et al also reported that there was no association between prepregnancy BMI of mothers and the development of AD in their children.^32^ Chen et al performed a meta-analysis for childhood AD and the mother’s BMI; they reported that the risk of AD increased in underweight mothers and decreased in obese mothers.^50^ A total of 96,265 patients were included in the meta-analysis; most of them were diagnosed with AD when they were older than 3 years. Therefore, the inconsistent results may be due to differences in disease definition from that in our study, which focused on children younger than 3 years of age.

There are several possible reasons why the effect of maternal prepregnancy BMI differs between asthma, FA, and AD, which is a risk factor for bronchial asthma, as it causes inflammation of the airways^51^ (which may be a risk factor for nonatopic asthma if it occurs *in utero*). Patel et al found that maternal prepregnancy BMI correlated with asthma prevalence only if the parents had no allergic disease.^52^ Leptin plays an important role in placental development, and the placenta also secretes leptin.^53^ An animal model shows expression of leptin receptor in fetal lung.^54^ Leptin is also involved in the maturation of epithelial cells in fetal alveoli,^55^^,^^56^ and early and excessive exposure to leptin *in utero* may affect lung maturation. Deficiency or excess of leptin could cause intrauterine growth retardation, which in turn could lead to lung damage such as chronic lung damage.^53^ These effects of leptin on fetal lung *in utero* lead to inappropriate airway sensitivity after birth. In addition, an experiment using a mouse model showed that loss of adiponectin activates macrophages in mouse alveoli, resulting in the development of emphysema-like lesions.^57^ The effects of leptin and adiponectin during airway development may contribute to the development of bronchial asthma, and a high prepregnancy BMI in mothers may result in an increased risk of asthma development in their children.

The strength of this study is in its large sample size and prospective design. Allergic diseases are relatively common, and we were able to collect data from a large number of patients. Maternal overweight and obesity were positively correlated with asthma and negatively correlated with FA, although the effect size is not very large. Because multiple factors influence the development of allergic diseases, the effect size of each factor is expected to be small.

Our study has several limitations. First, because our study was an observational study rather than an interventional study, various exposure factors may have been directly involved in the development of allergic diseases. Second, we determined the status of asthma, FA, and AD on the basis of questionnaires filled out by parents. FA can be categorized as IgE mediated and non–IgE-mediated depending on the underlying immunologic mechanism. Diagnosis of FA can be based on a combination of clinical history and laboratory data (specific IgE level and/or skin prick test result, as well as oral food challenge result).^58^ Therefore, distinguishing real FA from other diseases by using a questionnaire can be difficult. Finally, because the BMI of mothers is affected not only by fat but also by muscle mass, it would be desirable to use methods to measure fat mass, such as tricep skinfold measurement.

In summary, to the best of our knowledge, this is the largest cohort study to investigate the relationship between childhood allergy and maternal prepregnancy BMI. Our study confirmed that increased maternal BMI is a risk factor for the development of asthma in offspring. We also showed that an increase in mothers' prepregnancy BMI was negatively associated with the development of FA in their children. The effect of mothers' prepregnancy BMI on the development of allergic diseases in their children may vary depending on the disease. Because our study was conducted in a single country, further studies in other regions are required to confirm the broader applicability of the findings outside Japan.

**Clinical implications**

Children born to mothers with a high BMI are at a higher risk of developing asthma but are less likely to develop food allergies.

## Supplementary data

Table E1
