# Supplementary material for: Maternal BMI and allergy in children until 3 years of age (JECS)
Source: J Allergy Clin Immunol Glob. 2022 Mar 10;1(2):43–50. doi: 10.1016/j.jacig.2022.02.003 (PMC10510001; doi:10.1016/j.jacig.2022.02.003)
Supplement: Table E1 [file mmc1.docx]

Table E1. Number of children in each model

FA, food allergy; CMA, cow’s milk allergy; EA, egg allergy; AD, atopic dermatitis.

| BMI  (kg/m^2^) |  | | <18.5  No. of children (%) | 18.5-<25  No. of children (%) | 25-<30  No. of children (%) | 30=<  No. of children (%) |
| --- | --- | --- | --- | --- | --- | --- |
| Asthma | [model 1] | No | 9,552(89.2) | 43,801(88.9) | 4,202(87.0) | 1052(85.7) |
|  |  | Yes | 1,162(10.8) | 5,472(11.1) | 626 (13.0) | 175(14.3) |
|  | [model 2] | No | 9,443(89.2) | 43,387(88.9) | 4,154(87.0) | 1,041(85.7) |
|  |  | Yes | 1,148(10.8) | 5,421(11.1) | 622 (13.0) | 174(14.3) |
| FA | [model 1] | No | 9,332(87.1) | 42,708(86.7) | 4,307(89.2) | 1,104(90.0) |
|  |  | Yes | 1,382(12.9) | 6,565(13.3) | 521(10.8) | 123(10.0) |
|  | [model 2] | No | 9,226(87.1) | 42,303(86.7) | 4,260(89.2) | 1,092(89.9) |
|  |  | Yes | 1,365(12.9) | 6,505(13.3) | 516(10.8) | 123(10.1) |
| CMA | [model 1] | No | 10,380(96.9) | 47,519(96.4) | 4,709(97.5) | 1,206(98.3) |
|  |  | Yes | 334(3.1) | 1,754(3.6) | 119(2.5) | 21(1.7) |
|  | [model 2] | No | 10,260(96.9) | 47,070(96.4) | 4,658(97.5) | 1,194(98.3) |
|  |  | Yes | 331(3.1) | 1,738 (3.6) | 118(2.5) | 21(1.7) |
| EA | [model 1] | No | 9,882(92.2) | 45,231(91.8) | 4,516(93.5) | 1,160(94.5) |
|  |  | Yes | 832(7.8) | 4,042(8.2) | 312(6.5) | 67(5.5) |
|  | [model 2] | No | 9,767(92.2) | 44,800(91.8) | 4,466 (93.5) | 1,148(94.5) |
|  |  | Yes | 824(7.8) | 4,008(8.2) | 310(6.5) | 67(5.5) |
| AD | [model 1] | No | 9,464 (88.3) | 43,596 (88.5) | 4,298(89.0) | 1,088(88.7) |
|  |  | Yes | 1,250(11.7) | 5,677(11.5) | 530(11.0) | 139(11.3) |
|  | [model 2] | No | 9,357(88.3) | 43,186(88.5) | 4,250(89.0) | 1,077(88.6) |
|  |  | Yes | 1,234(11.7) | 5,622(11.5) | 526(11.0) | 138(11.4) |
